# The ‘Court of Public Opinion:’ Public Perceptions of Business Involvement in Human Rights Violations

**DOI:** 10.1007/s10551-022-05147-5

**Published:** 2022-07-04

**Authors:** Matthew Amengual, Rita Mota, Alexander Rustler

**Affiliations:** 1 Saïd Business School, University of Oxford, Park End Street, Oxford, OX1 1HP UK; 2 Centre for Corporate Reputation, Saïd Business School, University of Oxford, Oxford, UK

**Keywords:** Business and human rights, Public opinion, Multinational enterprises

## Abstract

Public pressure is essential for providing multinational enterprises (MNEs) with motivation to follow the standards of human rights conduct set in soft-law instruments, such as the United Nations Guiding Principles on Business and Human Rights. But how does the public judge MNE involvement in human rights violations? We empirically answer this question drawing on an original survey of American adults. We asked respondents to judge over 12,000 randomly generated scenarios in which MNEs may be considered to have been involved in human rights violations. Our findings reveal substantial gaps between public judgments and the standards set in soft law and the normative literature. We identify the attributes of episodes of human rights violations involving MNEs that influence public judgments, including the relationship between the MNE and the perpetrator, the practice of due diligence, and the type of abuse. These results provide insights as to when we might expect public pressure to drive MNE compliance with soft-law instruments, and they direct attention to specific standards that will likely require stronger, ‘hard’ law approaches or broader efforts to shift the public’s view.

Pressures on businesses to respect human rights emanate, in part, from the so-called ‘court of public opinion’ (Ruggie, 2008a). This is especially true in the case of multinational enterprises (MNEs), which operate across jurisdictions where human rights are afforded varying levels of protection. In the absence of an international treaty on business and human rights, a number of non-legally binding instruments have proliferated over the last decade. These ‘soft-law’ instruments reflect the widely accepted claim that businesses have human rights obligations, despite the ongoing debate on the exact nature of those obligations (Arnold, 2016; Wettstein, 2010a). The soft-law approach to business and human rights is grounded in the recognition of MNEs’ desire to preserve their reputation and ‘social license to operate,’ which can be negatively affected by public judgments that an MNE has been involved in human rights abuses (Arnold, 2010; Buhmann, 2016; Cragg, 2012; Muchlinski, 2001). It follows that public pressure, and, therefore, public views regarding human rights, fundamentally shape the environment in which MNEs operate. Where commonly held beliefs align with standards deriving from soft-law instruments and with perspectives in the literature that form the normative foundations of those instruments, one can reasonably expect MNEs to experience public pressure to act in accordance with these standards.^1^ Where they do not, there is a greater risk that noncompliance will carry few reputational consequences.

Yet, how the general public views MNE involvement in human rights violations is not well understood. The business and human rights literature has generated important insights on the existence and contours of business human rights obligations (Arnold, 2016; Bilchitz & Deva, 2013; Hsieh, 2015; Macdonald, 2011; Wettstein, 2010a), as well as on the changing regulatory landscape (Choudhury, 2018; Deva, 2021; Seppala, 2009; Wettstein, 2012a). But, while researchers have examined managers’ and employees’ perceptions of business human rights obligations (Arkani & Theobald, 2005; Egels-Zandén, 2017; Giuliani et al., 2020; McBeth & Joseph, 2005; Obara, 2017; Puncheva-Michelotti et al., 2010), few have addressed questions related to the views of the general public (Schrempf-Stirling & van Buren, 2020).^2^ In this paper, we ask: how does the public judge MNE involvement in human rights violations?

Our study brings a novel empirical approach to the business and human rights literature by deploying an original survey of a diverse national sample of 2420 American adults. The survey included a conjoint experiment that presented respondents with randomly generated vignettes. After each vignette, respondents indicated whether they perceived a focal MNE as being involved in a human rights violation. While all vignettes described situations in which an MNE could be linked to a human rights violation, they varied key elements of the episode that were selected in light of soft-law instruments and related normative debates in the business and human rights literature. By randomizing features of the vignettes, we are able to estimate the average causal effect of each element on public judgments, allowing us to answer key questions about the way members of the American public construe business human rights violations. Does the relationship between the MNE and the perpetrator influence public judgments? Do different types of rights provoke distinct responses from the public? Is the public sensitive to local (as opposed to internationally recognized) views of permissible behavior? How does the practice of due diligence influence public views? Does the public use an MNE’s industry or size to infer whether it has been involved in a human rights violation?

This paper makes an empirical contribution to the literature on business and human rights by answering these questions. Although MNE human rights conduct does not exclusively depend on public judgments, public opinion is nevertheless a central element in theories of human rights governance because companies are concerned about their reputations (Diermeier, forthcoming) and public opinion provides a political resource for activists (Soule, 2009). Yet, business and human rights research has been developed with little attention to when we might expect the public to be more or less likely to judge an MNE as being involved in a human rights violation. We make a unique contribution to the literature by identifying factors that shape public judgments of MNE human rights conduct, and by shedding light on the weight that each of those factors has on the public’s views of MNE involvement in human rights violations.

This knowledge is important for the business and human rights field for two reasons. First, it informs debates in the business and human rights literature, and amongst policymakers, regarding the right balance between ‘soft’ and ‘hard’ law approaches to the regulation of MNE human rights conduct (Augenstein, 2018; Parella, 2020). A better understanding of when the public is more or less likely to judge a company to be involved in a human rights violation facilitates the identification of specific standards that require stronger, ‘hard’ law approaches; equally, it can guide efforts to shift the public’s view, so that ‘soft’ law instruments become more effective as a result of increased social pressure for compliance.

Second, a robust understanding of how the public judges MNEs’ involvement in human rights violations can underpin a more rigorous debate on the benefits and shortcomings of relying upon a ‘business case’ for MNE compliance with human rights standards. Despite the popularity of instrumental approaches to corporate social responsibility, scholars have argued that it is unwise to assume that moral and instrumental reasons for action always converge in corporate settings (Demuijnck & Fasterling, 2016; Gond et al., 2009; Paine, 2000), and that this assumption is particularly dangerous with regard to human rights (Wettstein, 2012b). Our study demonstrates that, under certain circumstances, the public systematically discounts MNE involvement in human rights violations; if that happens, the ‘business case’ for compliance is likely weaker. A better understanding of public judgments of MNE involvement in human rights violations provides a way to identify areas of tension between economic and moral concerns (Margolis & Walsh, 2003), and demonstrates the need for the development of theoretical frameworks that take that tension into account.

In addition, our study furthers connections between business and human rights research and a growing body of literature on corporate social irresponsibility (Clark et al., 2021; Fiaschi et al., 2017; Kölbel et al., 2017; Lin-Hi & Müller, 2013; Nardella et al., 2020). Human rights violations are one particularly important type of corporate social irresponsibility (Giuliani et al., 2014), yet it is unclear whether theories of perceptions of irresponsible acts broadly defined (Lange & Washburn, 2012) generalize to this domain. Studies suggest that the general public blames buyers for the behavior of their suppliers (Hartmann & Moeller, 2014), that reputations spill over among corporations in the same industry (Barnett & King, 2008; Jonsson et al., 2009; Yu et al., 2008; Zavyalova et al., 2012), and that perceived severity of harms is key when people make attributions of irresponsibility (Lange & Washburn, 2012). Examining the assumptions and expectations of these theories in the context of human rights violations provides an opportunity to refine our understanding of perceptions of a highly consequential type of irresponsibility (Giuliani et al., 2014). Furthermore, a focus on business and human rights also foregrounds the potential for underexplored factors, such as due diligence, to influence public judgments of unethical behavior. Human rights due diligence is substantively different from other types of business due diligence because it focuses on risks to rights-bearers rather than risks to the firm, and it is meant to be an ongoing, iterative effort (Ruggie, 2009; United Nations, 2011). Hence, our analyses explore how public judgments are affected by proactive corporate behavior that demonstrates care, rather than by the mere sincerity of socially oriented gestures (Chen et al., 2020; Warren, 2022).

The paper proceeds by, first, situating our study in the business and human rights literature and highlighting the role of public opinion in the governance of MNE conduct. The following sections describe the empirical methods and report the main results. The paper then considers whether the findings generalize to people who are more politically active and, therefore, are more likely to exert pressure on MNEs. It concludes with a discussion of implications for the transnational governance of human rights.

## Social Expectations, Public Perceptions, and Human Rights

It is widely accepted that human beings have certain fundamental rights that ought to be respected. The existence and importance of human rights have been justified from a variety of philosophical perspectives (see, e.g., Nickel, 1987; Nussbaum, 1997; Shestack, 1998). While, historically, human rights were seen as a matter for states, it is now almost universally accepted that businesses also have human rights responsibilities. This acceptance stems from an acknowledgment of the power that businesses hold, as well as their impact on society. The precise nature and scope of business human rights responsibilities are still debated. While not rejecting the existence of related moral obligations, some authors resist the notion that private actors can be ascribed human rights obligations that are comparable to those of states (Bishop, 2012; Hsieh, 2015, 2017). Other authors defend the existence of such obligations, but suggest that they are fairly limited in scope (Arnold, 2010). Another growing strand of the literature argues that businesses have a responsibility not only to respect human rights, but also to protect and realize them under certain circumstances (Santoro, 2009; Wettstein, 2009; Wood, 2012).

Despite the debate on the nature and scope of business human rights responsibilities (for an overview, see Brenkert, 2016), it is broadly accepted that businesses have at least some human rights-related obligations. A growing field of scholarship has sought to understand how business human rights responsibilities can be, and are, discharged. Accordingly, the business and human rights field has expanded to include not just legal and philosophical accounts, but also contributions from other disciplines, such as management (Schrempf-Stirling & van Buren, 2020; Wettstein et al., 2019). This expansion reflects the recognition that the current legal framework is not sufficient to ensure business respect for human rights and that it is necessary to examine extra-legal factors that may feed into human rights governance.

Social expectations are one of the extra-legal factors of particular importance to MNE compliance with human rights standards, especially where domestic legislation is insufficient to address MNE behavior. This is because, in the absence of a legally binding treaty, soft-law instruments play a central role in establishing standards and governing MNE human rights conduct (Kirkebø & Langford, 2018). The success of these instruments relies heavily on monitoring and enforcement by non-state actors (Nolan, 2014; Ruggie & Sherman, 2017).

Among these initiatives, the United Nations Guiding Principles on Business and Human Rights (2011: the UNGP) is the most prominent. The UNGP and similar governance mechanisms are, at least implicitly, built on a ‘business case’ for compliance that derives from the public pressure MNEs experience when they are involved in human rights violations (Arnold, 2010; Carroll & Shabana, 2010). John Ruggie, who developed the UNGP, explicitly appealed to the link between social expectations, social license to operate, and MNE human rights conduct (Ruggie, 2008a, para. 54). According to Ruggie, the business responsibility not to violate human rights corresponds to a “transnational social norm” that “has acquired near-universal recognition” (Ruggie, 2009, para. 46; Ruggie & Sherman, 2017, p. 923), and failure to comply with that social norm “can subject companies to the courts of public opinion” (Ruggie, 2008a, para. 54). The phrase ‘court of public opinion’ has become a widely diffused metaphor for the reputational pressures experienced by corporations that are implicated in human rights violations.

Scholarship has identified a series of mechanisms through which the ‘court of public opinion’ plays a role in human rights governance. MNEs value their good standing and reputation (Diermeier, forthcoming), which is harmed when the public perceives them as being involved in a human rights violation (Wheeler, 2015). MNEs seek to maintain their social license to operate, which depends on support from the general public (Buhmann, 2016). Reputations also matter because consumers boycott products from MNEs that they perceive to be involved in human rights abuses (Kam & Deichert, 2020) and report a willingness to pay a premium for goods sold by firms that respect human rights (Hertel et al., 2009).

Public opinion also influences the work of activist groups that advocate for stronger human rights protections. Broad public support or opposition constitutes part of overall political, social, and corporate opportunity structures in national and international domains (Soule, 2009). Where public opinion is supportive, social movements are more successful (Soule & Olzak, 2004). Public opinion also influences the tactics of social movements that choose frames that they believe will resonate with the general public (Carpenter, 2005). The role that public opinion plays in influencing social activism is important because campaigns can influence firm reputations and financial performance (King & Soule, 2007).

In addition to playing a direct role in the governing of business conduct, public opinion influences efforts to create new business and human rights legislation. In the lead up to the 2017 French Duty of Vigilance Law, public awareness of pervasive corporate human rights misconduct accelerated efforts to pass legislation to enhance MNE accountability (Evans, 2020). Perhaps most directly, the 2020 Swiss Responsible Business Initiative, a proposal to hold MNEs accountable for their conduct abroad, failed to achieve a majority in a sufficient number of cantons in a referendum, limiting a potential ‘hardening’ of soft-law standards.

Despite the importance of public opinion in human rights governance, we know surprisingly little about how the general public judges the conduct of MNEs. Under what conditions is the public likely to believe that an MNE is involved in a human rights violation?

## Contextual Elements and Public Opinion

In this section, we discuss a set of contextual elements that may influence public opinion about MNE involvement in human rights violations, and that are prominent in soft-law instruments and in the normative literature. The contextual elements discussed in this section form the basis for our experimental design. We focus on MNE operations outside of the firm’s home country because this is the context of most policy and scholarship debates (Muchlinski, 2001; Ruggie, 2013; Wettstein, 2009). We group these elements in three broad categories: the type of MNE involvement, the nature of the abuse, and characteristics of the focal MNE.

### Type of involvement

We begin with the type of MNE involvement in a human rights abuse: specifically, the relationship between the MNE and the perpetrator, and whether and how the MNE undertakes human rights due diligence.

#### Relationship with the Perpetrator

The relationship between the focal MNE and the perpetrator constitutes a key feature of episodes of involvement in human rights violations. We define the ‘perpetrator’ as the actor that engages in the actual or potential abuse. MNEs are most commonly accused of being involved in human rights violations through the actions of their subsidiaries, suppliers, and governments of the states where they operate.

Under the UNGP, the responsibility to respect human rights falls upon the ‘business enterprise;’ although this term is not defined, it is usually interpreted as including both the parent company and its subsidiaries (Cassell & Ramasastry, 2016, p. 47). The Organization for Economic Cooperation and Development (OECD) Guidelines for Multinational Enterprises (2011: the “OECD Guidelines”), whose human rights chapter is aligned with the UNGP, explicitly apply to ‘enterprise groups,’ including subsidiaries. Research suggests that social evaluations of an MNE are affected by those of its subsidiaries (Zavyalova et al., 2012). Based on the tendency for the public to associate MNEs with their subsidiaries, we treat this type of relationship as a baseline with which to compare other relationships.

Many MNEs operate globally through networks of suppliers instead of subsidiaries. In doing so, MNEs add distance between themselves and the businesses that may be accused of human rights violations. While MNEs may not formally control suppliers, scholars have argued that MNEs still have human rights obligations in these circumstances (Macdonald, 2011) and activists have implicated MNEs in abuses perpetrated by suppliers. A prominent example is the scandal that arose in the 1990s around Nike. Nike outsourced production to Asian factories that violated international labor standards, including prohibitions on child labor. Despite Nike’s efforts to claim that they did not control suppliers, anti-sweatshop campaigns implicated Nike directly, leading the MNE to address human rights abuses by its suppliers (Locke, 2013).

The UNGP address the possibility of MNEs contributing or being directly linked to their suppliers’ abuses (Van Ho, 2021). Domestic legislation in a number of countries obliges MNEs to take (limited) steps to address potential human rights abuses in their supply chains, including the UK Modern Slavery Act. Political movements in many countries have sought to expand these legal obligations, drawing on public support generated by high-profile scandals (Evans, 2020).

Yet, evidence on the extent to which the public views MNEs as tightly linked to their suppliers’ human rights conduct remains incomplete. Work on corporate social irresponsibility suggests that perceptions of corporate culpability may be weakened if other actors can plausibly be seen as causally linked to the harm (Lange & Washburn, 2012). Yet, one study of the general public in Germany suggests that MNEs are perceived to be as responsible for their own environmental actions as for those of their suppliers (Hartmann & Moeller, 2014). Does the public perceive MNE involvement in human rights violations similarly if the offending business is a supplier instead of a subsidiary?

MNEs have also been accused of complicity with the human rights abuses of states in the countries where they operate. Unlike mere involvement, complicity requires knowledge that one’s actions or omissions might have harmful effects (Clapham & Jerbi, 2001). We focus on two types of complicity with states: beneficial complicity, which occurs when MNEs accrue benefits from the human rights impacts; and silent complicity, which refers to MNEs’ failure to speak out about those impacts (Tófalo, 2006; Wettstein, 2010a, 2013).

We begin with beneficial complicity, which is directly addressed in the Commentary to the UNGP (United Nations, 2011, p. 18). When an MNE knowingly benefits from a state’s actions to repress labor unions or to violently suppress protests, for example, it may be complicit in the violation (Wettstein, 2010a).

Even if they do not benefit from an abuse, businesses may be expected to condemn human rights abuses by others. Failure to do so may carry culpability (Clapham & Jerbi, 2001) due to the fact that silence can be interpreted as “moral support or encouragement” (Wettstein, 2010a, p. 37). Even though MNEs often attempt to portray themselves as ‘innocent bystanders’ in this type of situation, it is clear that their mere presence in an abusive context may equate to (if not legal, at least moral) complicity (Martin, 2011). The case of Shell in Nigeria is often used as an example of such silent complicity because the MNE remained silent when activists protesting oil exploration were executed without a fair trial. Shell’s silence and continuation of economic activities in the face of known human rights violations by the Nigerian state were widely interpreted as constituting tacit support for the state’s actions.

The number of allegations of business complicity is substantial (Ruggie, 2008b, c; Tófalo, 2006), yet we know of no analyses of the public’s view of them. Is the public as likely to judge an MNE to be involved in human rights violations when the relevant relationship is with a state, instead of a subsidiary or a supplier? Does the public differentiate between beneficial and silent complicity? Answers to these questions are key to understanding when MNEs will face more or less public pressure to comply with standards of human rights conduct.

#### Due Diligence

The public’s view of MNE involvement in human rights violations may also be influenced by whether a focal business is responsive to the context in which it operates and undertakes actions to address potential violations. Central to these actions is human rights due diligence: a standard of prudent conduct that includes conducting impact assessment, acting to address impacts, and monitoring the effectiveness of those actions (Ruggie & Sherman 2017). Under the UNGP, due diligence corresponds to an obligation to engage actively with human rights impacts; it is a standard of expected conduct that corporations must meet in order to discharge their responsibility to respect human rights (Ruggie, 2009; United Nations, 2011).

Stakeholders increasingly demand that MNEs undertake due diligence. For example, Oxfam criticized U.S. and European food retailers that source from suppliers at high risk of human rights violations without undertaking due diligence (Franck & Prapha, 2021). A failure to conduct due diligence may be perceived as a lack of care or concern for those affected. Studies have shown that, when people perceive corporate failure as something that could have been controlled, they tend to react negatively (Park & Rogan, 2019). Furthermore, businesses that perform impact assessments, but do not act on the findings, breach their obligations under the UNGP.

Conversely, if a firm identifies a human rights impact and tries, but fails, to prevent or mitigate it, the public may be much more forgiving. Under the UNGP, an MNE that conducts human rights impact assessment, identifies an actual or potential harm, and takes appropriate action to mitigate that impact, is acting in accordance with its obligations. Even the best efforts may fail, and the public is likely to understand this. There is evidence that people are more lenient when an actor fails to achieve a certain outcome if that actor put in high levels of effort; in such cases, people are more likely to respond with sympathy (Weiner, 1995). Furthermore, visible attempts to prevent or mitigate harm may be perceived as manifestations of care, which is likely to weaken damaging perceptions of hypocrisy (Chen et al., 2020).

This analysis suggests another series of pertinent questions that inform our understanding of whether public pressure may contribute to improving MNE human rights conduct. Are people less likely to perceive an MNE to be involved in human rights abuses when it conducts due diligence? Are people less likely to perceive an MNE as being involved in human rights violations if the MNE unsuccessfully acted on findings than if the MNE did not act, or did not conduct due diligence at all?

### Nature of the Abuse

Although prominent international organizations, such as the UN, take a broad approach to defining the spectrum of human rights that businesses have a responsibility to respect, we do not know how the public views this spectrum. We consider different types of abuses that affect a variety of human rights, as well as how information on local norms regarding specific abuses may influence public judgments of MNEs.

#### Abuses

Although the UN framework (2011) asserts that businesses should respect, at a minimum, all rights included in the International Bill of Human Rights and the Declaration on Fundamental Principles and Rights at Work set out by the International Labor Organization (ILO), the range of human rights that businesses consider in practice varies widely. For example, a study of FTSE 100 firms found that, while 77% of them included the right to form a trade union, only 18% included the right to a decent standard of life (Preuss & Brown, 2012). If large swaths of the public do not recognize a certain human right, they will not judge an MNE linked to impacts on that right to be involved in a human rights violation. In this subsection, we discuss six representative abuses that we include in our empirical study.

We begin with child labor, one of the most widely referenced abuses in the context of business and human rights. Child labor violates rights that are recognized in a large number of policy instruments, such as the Universal Declaration of Human Rights (UDHR) and the Convention on the Rights of the Child (CRC). Child labor is a highly emotive issue (White, 1994), which attracts public attention in the Global North like few other issues occurring in the Global South (Edmonds, 2007). The public’s magnitude of sympathy, and likelihood to deem a particular human rights abuse as severe, is heightened if the victims are considered to belong to an especially vulnerable group, such as children (Diermeier, forthcoming). Given the broad legal recognition of the rights involved, as well as the moral outrage that the abuse is likely to cause, we expect child labor to trigger strong responses regarding MNE involvement; we therefore treat this abuse as a baseline.

Many allegations of business human rights abuses are related to other attributes of workers’ rights. Among the wide spectrum of workers’ rights, the right to a living wage is particularly salient. The right to a just and favorable remuneration, that allows workers and their families to have an adequate standard of living, is enshrined in the UDHR and the International Covenant on Economic, Social, and Cultural Rights (ICESCR). Beyond the requirement that businesses have to pay their own workers adequately, scholars have argued that buying from suppliers that do not pay a living wage can, in and of itself, constitute a human rights abuse (Giuliani et al., 2014). Rights organizations have pushed to include living wages in the human rights obligations of MNEs leading global value chains (LeBaron et al., 2021).

There is evidence, however, that businesses do not subscribe to such a demanding view of their obligations regarding living wages. For example, Obara and Peattie (2018) found that many firms view their human rights obligations as largely restricted to the domain of ‘doing no harm,’ which arguably does not preclude the payment of sub-living wages in supply chains. Only some businesses have committed to living wages, and many of those that have made such commitments have been criticized for failing to deliver (LeBaron et al., 2021).

Evidence from a 2009 study suggests that a majority of Americans perceive the right to a minimum standard of living as a human right (Hertel et al., 2009). More recently, intensive campaigns around living wages, including those linked to the economic effects of the Covid-19 pandemic, may have expanded these views further. Yet, we do not know how the general public assesses situations where an entity related to an MNE fails to pay a living wage.

A further type of human rights abuse that has become increasingly visible is the discrimination of ethnic minorities. This type of abuse violates rights affirmed in a variety of international human rights instruments, including the UDHR, the International Covenant on Civil and Political Rights (ICCPR), the ICESCR, the CRC, and the UN Declaration on the Rights of Persons Belonging to National or Ethnic, Religious and Linguistic Minorities. In an employment context, discrimination against ethnic minorities breaches ILO Convention No. 111. While the UNGP do not explicitly address racial discrimination, their reference to international human rights instruments that do address this issue should be read as implicit recognition of this type of violation (George, 2021). Discrimination of ethnic and racial minorities in business practices, such as hiring, is still pervasive across the world, despite domestic and international legal protection (Quillian et al., 2019). Social movements calling attention to racism and discrimination have grown in recent years and may influence the public’s view. Yet, there is evidence that ‘racial apathy’ is an increasingly common form of prejudice; this phenomenon could desensitize the public to some extent (Forman & Lewis, 2006), making it less likely that people perceive discrimination as a human rights issue. It is therefore unclear how the public judges MNEs that are related to perpetrators engaged in this type of abuse.

Violent repression of protests is another human rights abuse that is frequently found in business contexts (Del Bene et al., 2018, p. 620; Poulos & Haddad, 2016, p. 5; Wettstein, 2010a, p. 36, 2012a, p. 742). The violent repression of protests can violate a number of human rights, such as the right to physical integrity, the right to liberty and security, the right to freedom of opinion and expression, and the right to freedom of peaceful assembly, as recognized in the UDHR and the ICCPR. The combination of physical injury and deprivation of freedom of expression is likely to trigger strong emotional reactions from the public. However, we do not know how the public views MNEs that are related to perpetrators of this type of abuse.

An additional type of human rights abuse that is pervasive in business contexts relates to environmental harm. A common example of environmental harm is the contamination of community land, which impacts, at a minimum, the right to live in a safe, clean, healthy, and sustainable environment; this right has been the focus of the UN Special Rapporteur on Human Rights and the Environment since 2012 (United Nations, 2019). Environmental rights are recognized in regional instruments, such as the African Charter on Human and Peoples’ Rights and the Inter-American System, but adjudicators dealing with environmental issues in other contexts often have to rely on the ‘greening’ of other human rights norms, such as the right to health. The ‘greening’ approach conceptualizes a safe, clean, healthy, and sustainable environment as essential to the full enjoyment of other internationally recognized human rights (United Nations, 2018). However, efforts to link the environment and human rights are fairly recent and underdeveloped. It is possible that many people still see them as separate themes, and, if that is the case, they may not be as likely to perceive MNEs linked to environmental damage as being involved in a human rights violation.

Our last representative example is the destruction of a sacred site, which harms cultural rights. Violations of cultural rights in business contexts often involve harm to indigenous rights, which are grounded in fundamental human rights principles such as non-discrimination, self-determination, and cultural integrity; these rights are recognized in specific instruments, such as the ILO Convention No. 169 and the 2007 UN Declaration on the Rights of Indigenous Peoples. A growing number of high-profile events, especially in the mining industry, has reinvigorated the public’s attention to cultural and indigenous rights. For example, mining MNE Rio Tinto destroyed Juukan Gorge, an Aboriginal sacred site in Australia, despite the opposition of the Puutu Kunti Kurrama and Pinikura peoples (Albeck-Ripka, 2020). Although these are severe incidents that often trigger public outcry, cultural rights remain relatively underdeveloped, for reasons that include the widespread view that culture is a ‘luxury’ (Stamatopoulou, 2007, pp. 4–6). They also may not trigger the same type of emotive response of moral outrage as other abuses, such as child labor.

While all of these rights are recognized in international law, we do not know how the public perceives situations where they are violated. How does the type of abuse affect the likelihood that the public will judge the MNE to be involved in a human rights violation?

#### Local Views

Beyond the type of abuse, public perceptions may also be influenced by local norms that differ from international human rights standards. That is, the public may tolerate behavior that constitutes a human rights abuse under international human rights law, but that is permissible in a particular local context. This is a matter of debate in the normative literature; hence, we root our inquiry into local norms in philosophical arguments regarding the universality of rights and of moral standards. The debate on the universality of rights is important because it points to arguments as to whether or not it makes sense to view human rights as applicable in every context around the world; more broadly, it is important to ask whether it is plausible to expect the same moral standards to apply to every actor, in every community.

A school of thought known as communitarianism rejects the notion that there can be universal human rights; according to this view, considerations of justice only make sense in the context of a particular community, with its shared history and shared meanings (MacIntyre, 2007, p. 69; Ulrich, 2008, p. 239). Universalism, on the other hand, claims that moral rights, based on the inherent worth and equality of every person, are universally valid (Donnelly, 2013; Werhane, 1985; Wettstein, 2009). Universalism is sometimes associated with concerns about moral imperialism and cultural assimilation; as a response to those concerns, other approaches attempt to combine elements of universalism and communitarianism. For example, Donaldson and Dunfee (1994) posit the existence of two different levels of moral norms: universal ‘hypernorms,’ deriving from a hypothetical macrosocial contract; and community-specific ‘microsocial contract norms,’ deriving from a ‘moral free space’ that allows communities to stipulate their own ethical rules, within the bounds of the ‘hypernorms.’ Microsocial contract norms must, however, be compatible with hypernorms, which the authors take to include ‘core human rights’ and the obligation to respect human dignity (Donaldson & Dunfee, 1994, p. 267). Similarly, Donnelly’s (1984, 2013) notion of ‘relative universality’ of human rights allows variation from context to context, so long as a number of universal rights are protected. The idea that there should be at least a degree of tolerance for local norms and traditions is likely to attract some sympathy from the public. This analysis raises the question: is the public less likely to perceive an MNE as being involved in a human rights violation if the abuse is permissible under local norms?

### Business Characteristics

The characteristics of the MNE are the final features that we investigate. We focus on the size and power of the MNE, as well as its industry.

MNE size and power may influence moral judgments regarding business human rights obligations (Clapham & Jerbi, 2001; Wettstein, 2010b; Wood, 2012); this claim is a manifestation of the widely held position that ‘ought implies can.’ A large company that is perceived to have power to influence the actions of a subsidiary, suppliers, or a host state can be perceived to have a moral obligation to intervene in the event of a human rights violation. Conversely, a small company may be judged not to have that power; hence, it may not be ascribed the same level of moral responsibility (Kim et al., 2015). Yet, we have a poor understanding of whether these factors are reflected in public perceptions. Does the size of the MNE affect public judgments about MNE involvement in human rights violations?

Finally, the industry in which an MNE operates may come to bear on public opinion. Organizations in stigmatized categories are stereotyped and viewed as essentially flawed; their actions are therefore interpreted in a negative light (Devers et al., 2009). Indeed, even in non-stigmatized categories, backlash for business misconduct and scandals ‘spill over’ among businesses in the same industry (Barnett & King, 2008; Yu et al., 2008; Zavyalova et al., 2012) as observers generalize from unethical behavior of one organization to other similar organizations (Jonsson et al., 2009). Are members of the public more likely to view an MNE as being involved in human rights abuses if it operates in an industry with a more salient history of abuse?

## Research Design

To answer these questions, we fielded an original survey of 2420 American adults. Before fielding the survey, we registered our design and analysis plan.^3^ The survey was completed between March and April 2021.^4^ We have chosen to focus on Americans because they are more likely to have power to influence corporate choices than less empowered communities in developing countries. The respondents were provided by the survey firm Dynata (formally, Survey Sampling International), which maintains a pool of respondents that is representative of the U.S. adult population in terms of gender, age, region, and other attributes. Dynata directed a sample from this pool to our online survey hosted on Qualtrics.^5^

Studies have shown that survey experiments conducted using online convenience samples provide results similar to those obtained using probability samples (Berinsky et al., 2012; Coppock, 2019; Mullinix et al., 2016). Our sample is more closely representative of the U.S. population than other commonly used convenience samples, such as Amazon Mechanical Turk, which often are substantially younger and more educated than the U.S. adult population (Berinsky et al., 2012). As shown in Table 1, our sample closely matches the U.S. adult population across a range of dimensions, with only a small difference in the portion of people who did not complete a high school education and who identify as Hispanic.

**Table 1:** Sample characteristics

	Sample (*N* = 2420) (%)	U.S. population over 18^a^ (%)
Age		
18–24	11.5	11.4
25–34	15.7	17.8
35–44	17.6	16.4
45–54	14.9	15.8
55–64 years	17.6	16.8
65 years and over	22.7	21.7
Education		
No high school diploma	6.8	10.9
High school or equivalent	30.3	28.6
Some college, less than 4-year degree	29.6	28.2
Bachelor's degree or higher	33.3	32.3
Race and ethnicity^b^		
White alone	65.8	62.9
Black or African American alone	12.4	12.0
Hispanic	13.0	16.6
American Indian and Alaska Native alone	0.8	0.8
Asian alone	6.2	6.0
Native Hawaiian and other Pacific islander alone	0.3	0.3
Two or more races	1.5	1.4
Gender		
Male	46.9	48.4
Female	52.9	51.6
Another gender identity	0.3	–

^a^Education, race, and gender from the 2018 current population survey. Age from the 2020 current population survey. We do not weight our sample

^b^There was one non-response for the race and ethnicity question. Therefore, the *N* for this question is 2419

We used a conjoint survey experiment that presented respondents with a set of randomly generated vignettes. Empirical researchers often embed experiments into large-scale surveys in order to test causal claims in samples that are representative of populations (Mutz, 2011). Using a survey experiment, we are able to answer the questions raised above about factors that may cause people to perceive that an MNE has, or has not, been involved in a human rights violation.

Conjoint experiments are one class of survey experiments that involve the simultaneous manipulation of the multiple elements of vignettes (for a detailed account, see Hainmueller et al., 2014). They have been increasingly used across social science fields to analyze a range of multidimensional public views, including perceptions of political candidates (Hainmueller et al., 2014), immigrants (Hainmueller et al., 2015), terrorist attacks (Huff & Kertzer, 2018), and climate policies (Bechtel & Scheve, 2013).

Conjoint experiments have characteristics that are helpful to answer the questions raised in the previous section. First, the judgments that people register in conjoint experiments have been shown to reflect real-world behavior (Hainmueller et al., 2015). Therefore, findings from these experiments are likely to be externally as well as internally valid. Second, by simultaneously randomizing multiple elements of vignettes, conjoint experiments can incorporate a wide range of treatments. This attribute is important in our setting because people’s judgments about MNE involvement in human rights violations depend on a number of contextual factors. With a more traditional survey experiment, we would only be able to probe a small number of these potential factors, while with a conjoint approach we can investigate a range of relevant factors in a single survey. Third, conjoint experiments can reduce social desirability bias (Horiuchi et al., 2021). Like most survey experiments, conjoint experiments do not require the respondent to state their views directly. Furthermore, conjoint experiments mix sensitive elements, such as people’s views of child labor, with non-sensitive elements. As a result, respondents are able to rationalize their judgments based on non-sensitive elements of the vignette and are less likely to perceive that their judgments will be seen by researchers as inconsistent with social norms.

In our survey, respondents were first given instructions, and then presented with five different vignettes, generating data on a total of 12,100 respondent judgments (a full transcript of the survey has been posted in our pre-registration).^6^ Each vignette contained six elements (Table 2), within the limits of the number of elements that have been shown to be effective in conjoint designs (Bansak et al., 2021).^7^ These elements were randomly combined to create a short narrative, so that, for each participant and for each round, the vignettes were different combinations of the elements described in Table 2.

**Table 2:** Structure of the vignettes

Attribute	Wording
*Size*	
Large	[Company X] is a large company with a lot of influence in the foreign country
Small	[Company X] is a small company that is just starting out in the foreign country
*Industry*	
Oil	Oil
Clothing	Clothing
Automobile	Auto manufacturing
Solar energy	Solar energy
Bicycle	Bicycle manufacturing
*Relationship*	
Subsidiary	[Company X] owns and controls a subsidiary business
Supplier	[Company X] buys inputs from a supplier in this foreign country
Beneficial complicity	[Company X] benefitted when this country’s government
Silent complicity	[Company X] did not speak out publicly to condemn this country's government
*Abuse*	
Employ child labor	Employ children
Pay less than living wage	Pay workers less than a living wage
Contaminate land	Contaminate a community’s land
Destroy sacred site	Destroy a sacred site
Violently repress protest	Violently repress protests
Discriminate against minorities	Discriminate against an ethnic minority
*Local view*	
Should not	People in this country think that [the government/businesses] should not be able to [abuse]
Can sometimes	People in this country think that [the government/businesses] should sometimes be able to [abuse]
*Due diligence*	
No due diligence	[Company X] did not try to find out whether [its subsidiary/its supplier/the government] might [abuse]
No action	[Company X] knew that [its subsidiary/its supplier/the government] might [abuse] but did nothing to prevent it
Tried to prevent	[Company X] knew that its subsidiary/its supplier/the government] might [abuse] and tried to prevent it

**Fig. 1:**
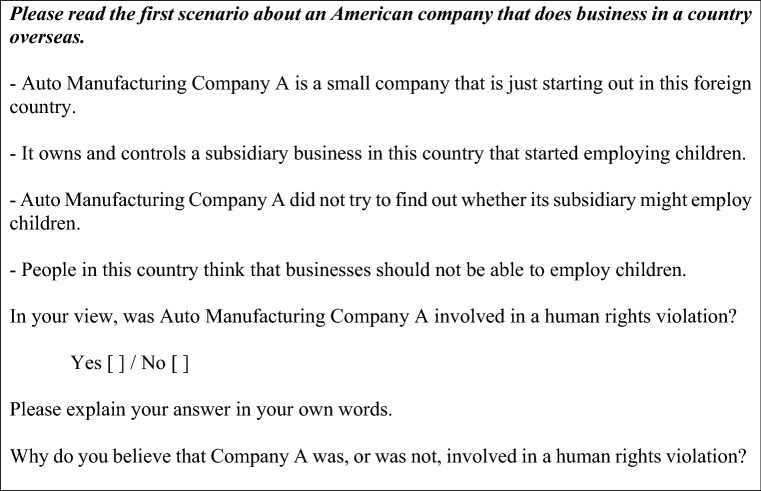
Example vignette

Figure 1 shows an example vignette.^8^ The vignettes described the MNE’s industry, which we selected to vary saliency of human rights abuses, measured for each industry by counting the number of articles and reports in English archived in the Business and Human Rights Resource Centre database. We chose high and low salience energy industries (oil, 8798 articles^9^; and solar energy, 39), as well as high, medium, and low salience manufacturing industries (clothing, 3832 articles; automobile manufacturing, 1366; and bicycle manufacturing, 8). The vignettes also included the size and level of influence of the MNE, as well as the relationship between the focal MNE and the perpetrator, the type of abuse, the way people in the country view the abuse, and information on any due diligence carried out by the focal MNE.

After each vignette, we asked respondents: “In your view, was Company X^10^ involved in a human rights violation?” We chose this wording because ‘involvement’ is a broad term (unlike ‘responsible,’ which can imply causality) and it is the type of judgment that MNEs concerned about public pressure seek to avoid. Respondents were given a simple Yes (1) / No (0) choice to answer the question. A binary choice simplifies the task and enables straightforward interpretation of the results. For the first two vignettes, respondents were also asked to explain their answer in their own words. We only asked open-ended questions for two of the five vignettes to reduce the burden on respondents. Of the 2420 participants, we randomly sampled 500 respondents and inductively hand coded 1000 responses to the open-ended questions (coding guide in Appendix, Sect. Open-Ended Responses).^11^

**Fig. 2:**
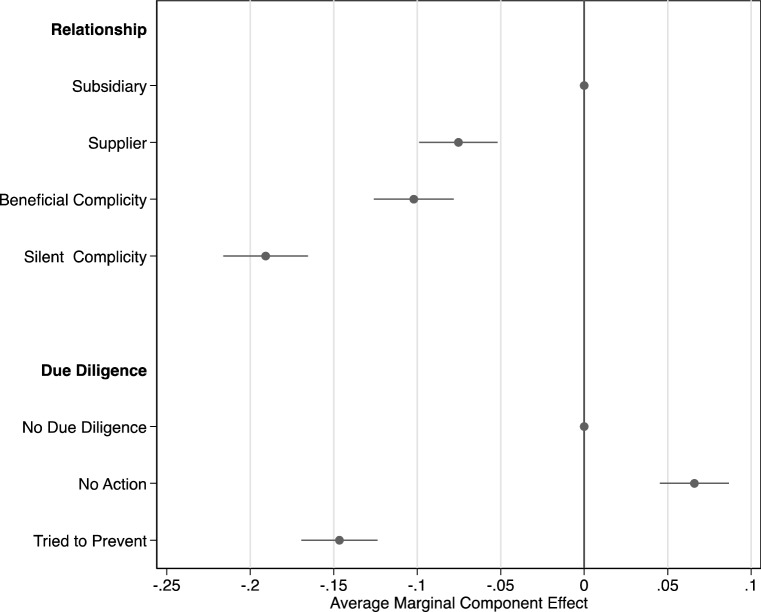
Type of involvement

**Fig. 3:**
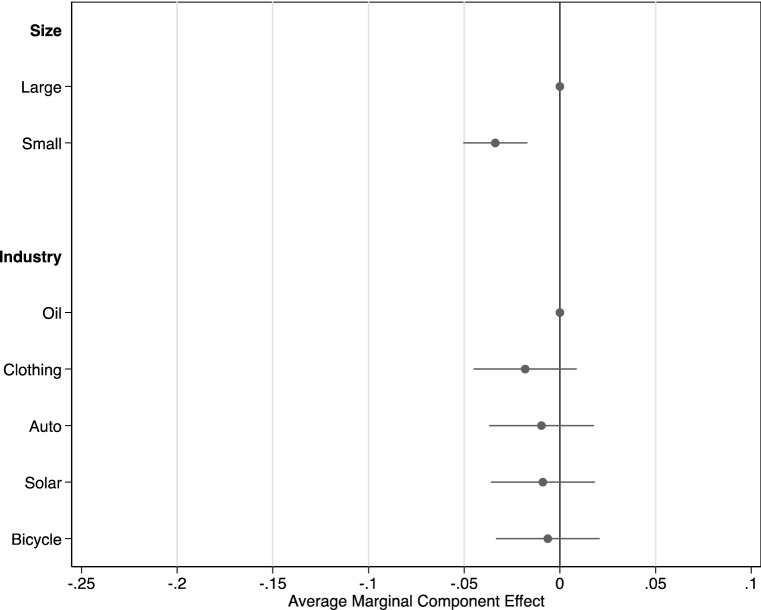
Focal firm characteristics

**Fig. 4:**
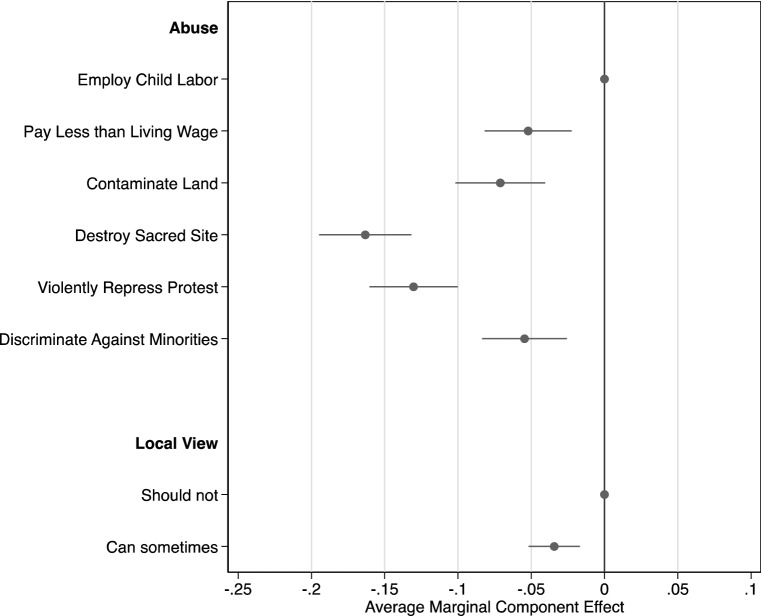
Abuses

**Fig. 5:**
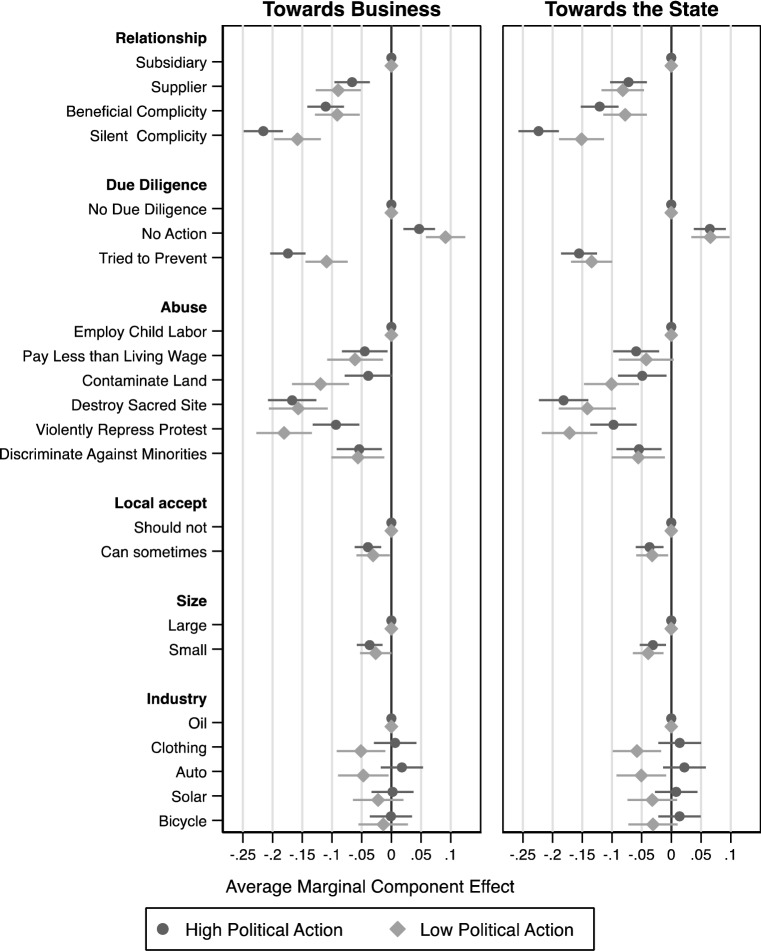
Political action

To understand which elements influence public judgments, we estimate the average marginal component effect (AMCE) of each element. Hainmueller et al. (2014) establish that the AMCE is the quantity of interest in conjoint experiments that analyze multidimensional preferences. To estimate the AMCEs, we follow standard practice and use an Ordinary Least Squares regression. We report the regression we used to estimate AMCEs in table form in the Appendix (Table 5). We also plot the AMCEs with 95% confidence intervals to ease interpretation (Figs. 2, 3, 4, 5). Each observation in these analyses is a response to a randomly generated vignette. The dependent variable is whether the respondent indicated that the MNE was involved in a human rights violation. The independent variables are dummy variables for each element of the vignette that the respondent saw. The coefficients of the regression are the AMCEs. They can be interpreted as the average causal effect of each element, conditional on the joint distribution of all other elements. Each AMCE is presented compared with a baseline (the omitted category in the regression). For example, the AMCE for ‘supplier’ is the average change in the probability of a respondent judging the MNE to be involved in a human rights violation when the perpetrator is a ‘supplier’ instead of a ‘subsidiary,’ conditional on the uniform distribution of all other attributes (i.e., size, industry, abuse, due diligence, and local views). Thus, by estimating the AMCEs we are able to answer questions regarding the influence of multiple contextual elements on American adults’ judgments of MNE involvement in human rights violations.

Finally, while our inferential goal is to understand the views of all American adults, public pressure on MNEs arises mainly from politically active individuals. To identify this subset of the population, we asked two blocks of questions that probed levels of political activity. One block asked about political activities towards business, another block asked about political activities towards the state (see Appendix Table 7). By asking these questions before the vignettes, we ensured that the answers would not be influenced by the treatments. We examine heterogenous treatment effects by estimating Average Component Interaction Effects (ACIEs), which can be interpreted as the difference between the AMCEs for each subgroup.

## Main Findings

Overall, respondents viewed the MNE as being involved in a human rights violation in 59% of all responses. This result is important in itself. The scenarios were constructed in line with prominent soft-law instruments and with influential normative arguments in the business and human rights literature; they were designed so that, in all cases, the MNE would have been involved in a human rights violation in light of those standards and arguments. However, in nearly four out of ten cases, the public did not judge the MNE as being involved in a human rights violation. The remainder of this section presents analyses of our findings regarding the different types of MNE involvement, MNE characteristics, and specific abuses, as well as an analysis of a subgroup of more politically active respondents.

### Type of Involvement

We begin with the effects of relationships and due diligence, reported in Fig. 2. Compared to the baseline of ‘subsidiary,’ vignettes that described the perpetrator as a ‘supplier’ caused respondents to be 7.5 percentage points less likely to perceive the MNE as being involved in a human rights violation (*p* < 0.001, 95% CI [− 0.099, − 0.052]). This represents a substantial decline in perceptions of involvement, which is surprising given both previous research (Hartmann & Moeller, 2014) and the efforts of activists to tie MNEs to the abuses of their suppliers.

Similarly, vignettes that described the MNE as benefitting from the actions of a government caused a 10.2 percentage point decline in the probability of people perceiving the MNE as being involved in a human rights violation, compared with ‘subsidiary’ (*p* < 0.001, 95% CI [− 0.13, − 0.078]). This effect is more negative than the effect of ‘supplier’ (*F*(1,2419) = 4.62, *p* = 0.032). Vignettes with ‘silent complicity’ caused respondents to be 19.1 percentage points less likely to perceive the MNE as being involved in a human rights violation (*p* < 0.001, 95% CI [− 0.22, − 0.17]). The effect of ‘silent complicity’ was substantially more negative than the effects of ‘supplier’ (*F*(1,2419) = 81, *p* < 0.001) and ‘beneficial complicity’ (*F*(1,2419) = 48, *p* < 0.001). These results demonstrate that proximity weighs heavily on public judgments: notwithstanding arguments that MNEs have obligations to address human rights abuses by suppliers and host states, the public strongly discounts these more distant relationships.

We now turn to due diligence. Compared with the baseline of ‘no due diligence,’ when vignettes stated that the MNE learned about potential abuses but did not take any action, respondents were 6.6 percentage points more likely to judge the MNE as being involved in a human rights violation (*p* < 0.001, 95% CI [0.045, 0.087]). When vignettes described the MNE as trying to prevent the abuse, people were 14.7 percentage points less likely to perceive involvement (*p* < 0.001, 95% CI [− 0.17, − 0.12]). The magnitude of this effect is substantial and demonstrates that attempting to prevent abuses shifts perceptions, even if those attempts are ultimately unsuccessful.

To understand the reasoning underlying these effects more fully, we draw on the open-ended responses. We inductively coded the justifications and uncovered four distinct reasons given that pointed to elements of the type of MNE involvement. Table 3 reports the frequency of each type and provides an example response. The most common type of justification linked to involvement emphasized responsiveness (38.7%). As shown in the example, respondents explained their judgments in terms of the actions that the MNE did, or did not, take in the context of potential abuses. The frequency of this form of reasoning reinforces our findings on due diligence and reveals the salience of mitigating efforts by an MNE for public judgments of its conduct.

**Table 3:** Justifications related to type of involvement

Code	Examples	% Responses	Responses with code (mean DV)	Responses without code (mean DV)	*p*
Responsive	“This company tried to do the ethical thing and protect the community and the environment by attempting to block the government from doing damage. No violations were committed”	38.7	0.64	0.61	0.47
Association	“It benefitted from discrimination which means they took part in said discrimination. Ergo they violated human rights”	7.7	0.69	0.62	0.24
Causal	“They did not directly employ the kids…”	10.1	0.51	0.64	0.03
Appropriate	“It is not that company’s job to take political action when they themselves are not violating anybody’s rights. A small clothing company is not responsible for the government’s actions”	3.3	0.00	0.64	< 0.01

Percentage of codable responses. *N* = 848. Responses could have multiple codes. *p-*value from a test of the difference in means of the DV between responses with the code, and those without

Beyond responsiveness, we find that 7.7% of responses were justified by pointing to the mere association (or lack of association) between the MNE and the perpetrator. Those using a logic of association were just as likely to judge the MNE as being involved compared with those that did not use this reasoning. By contrast, we find that 10.1% of answers indicated that respondents assessed involvement in causal terms, explicitly stating their inference about whether or not the MNE caused the abuse to justify their responses. Responses that expressed a causal criterion judged the MNE as being involved in a human rights violation 13 percentage points less often than when causal justifications were not present. These results suggest that the extent of individuals’ use of each of these criteria constitutes a key element of the way we can expect the public to respond to MNE conduct.

Finally, some respondents stated that it was inappropriate for MNEs to be considered involved in a human rights violation because at least some human rights, in their view, were the purview of states, not business (3.3%).

### MNE Characteristics

Examining characteristics of the focal firm, reported in Fig. 3, we find only a very modest effect of the size and power of the MNE. Vignettes that described the MNE as ‘small’ and ‘just starting out’ resulted in a 3.4 percentage points decrease in the probability that respondents judged the MNE to be involved in a human rights violation, compared with those that described the company as ‘large’ and having ‘a lot of influence’ (*p* < 0.001, 95% CI [− 0.051, − 0.017]). We further examine if the effects of distinct relationships and due diligence are larger or smaller depending on MNE size and power. We do not find that size conditions the ‘supplier’ (ACIE =  − 0.15, *p* = 0.54, 95% CI [− 0.062, 0.032]), ‘beneficial complicity’ (ACIE =  − 0.035, *p* = 0.15, 95% CI [− 0.081, 0.012]), or ‘silent complicity’ treatments (ACIE =  − 0.032, *p* = 0.19, 95% CI [− 0.080, 0.016]). Similarly, we do not find that size conditions the ‘no action’ (ACIE = 0.024, *p* = 0.24, 95% CI [− 0.16, 0.065]) or ‘trying to prevent’ treatments (ACIE = 0.022, *p* = 0.30, 95% CI [− 0.020, 0.064]). Therefore, the large effects that we find on the relationship and due diligence elements were similarly present in vignettes with large *and* small MNEs.

Contrary to our expectations, we did not find significant effects of MNE industry on perceptions of MNE involvement in human rights abuses. There were no statistically significant differences between vignettes that included an oil company and those that included a solar company. Similarly, there were no differences among the three manufacturing sectors (see Appendix Table 6 for all comparisons). This result suggests that people do not make strong inferences about a firm’s involvement based on the track record of its broader industry.

### Abuses

Figure 4 reports the effects of the different types of abuse and of the local views on those abuses. First, we find that vignettes that included the destruction of a sacred site were the least likely to be considered cases of involvement in human rights violations, compared with all other abuses (see Appendix Table 6 for all pairwise comparisons). The effect sizes were large: vignettes with ‘destruction of a sacred site’ were 16 percentage points less likely to be perceived as cases of MNE involvement, compared with vignettes that included ‘child labor’ (*p* < 0.001, 95% CI [− 0.19, − 0.13]). Second, we find that MNEs were 13 percentage points less likely to be perceived to be involved in a human rights violation when vignettes included ‘violent repression of protests,’ as opposed to ‘child labor’ (*p* < 0.001, 95% CI [− 0.16, − 0.10]). After the destruction of a sacred site, violent repression of protests resulted in the least probability of respondents perceiving MNE involvement. This result is surprising because such an abuse involves both physical injury and deprivation of freedom of expression.

Third, we find that ‘failure to pay a living wage’ (*b* =  − 0.052, *p* = 0.001, 95% CI [− 0.082, − 0.022]), ‘contamination of a community’s land’ (*b* =  − 0.071, *p* < 0.001, 95% CI [− 0.10, − 0.04]), and ‘discrimination against an ethnic minority’ (*b* =  − 0.055, *p* < 0.001, 95% CI [− 0.084, − 0.026]) all resulted in people being less likely to perceive the MNE as being involved in a human rights abuse, compared with ‘child labor.’ However, the negative effects were smaller than those for ‘violent repression of protests’ or ‘destruction of a sacred site.’

Turning to the local views of the abuse, we find only a modest effect. When vignettes indicated that people in the country believed that a business or government can sometimes take such an action (and, so, that the relevant behavior was permissible under local norms), it caused respondents to be 3.4 percentage points less likely to judge that the MNE was involved in a human rights abuse (*p* < 0.001, 95% CI [− 0.052, − 0.017]).

These results are striking, considering that all of the abuses included in the vignettes are violations of internationally recognized human rights. Furthermore, given that these effects are conditional on the other elements of the vignettes, including the relationship, it is notable that the type of abuse shifts judgments of MNE involvement so strongly, and so much more than local views regarding the permissibility of the behavior. To better understand the reasons underlying these results, we turn once again to the responses to the open-ended questions (Table 4). We inductively coded these responses and uncovered a set of recurring justifications related to the nature of the abuse. By far, the most common justifications described the categorical fit of an abuse with the respondent’s understanding of human rights. These responses simply stated that a particular abuse was, or was not, a human rights violation, without providing additional elaboration (19.1%). Much less often (6.4%), people referred to what they believed to be the content of legal norms—stating that some action was or was not illegal and, therefore, was, or was not, a human rights violation. The relative scarcity of legal references suggests that the law is not a highly salient reference point for the public when considering human rights. It also suggests an explanation for why scenarios with abuses that violated cultural rights are so strongly discounted by the public, even though these rights are recognized in international treaties (further analysis of codes by abuse in Appendix Table 8).

**Table 4:** Justifications related to abuses

Code	Example	% Responses	Mean DV with code	Mean DV without code	*p*
Categorical fit	“Destroying a sacred site does not involve human rights”	19.1	0.62	0.63	0.91
Legal reference	“Using kids as employees is illegal”	6.4	0.72	0.62	0.13
Deontological	“Discrimination is wrong, and should not be tolerated”	14.5	0.85	0.59	< 0.01
Consequential	“They are endangering the lives of people by letting contaminants go into areas that sustain life and are willing to let the contaminants damage the ecosystem”	3.9	0.87	0.61	< 0.01
‘American’ values	“Company A may not be in violation in that country but as an American company it is”	1.2	1.00	0.62	< 0.01
Local norms	“If the people are aware of this practice and appear to accept it then the oil company was acting on the approval of the country's government. No violations took place”	5.2	0.39	0.64	< 0.01

Percentage of codable responses. *N* = 848. Responses could have multiple codes. *p-*value from a test of the difference in means of the DV between responses with the code, and those without

Deontological justifications that associated human rights with universally applicable moral standards were also common in the responses (14.5%). These respondents perceived MNE involvement in a human rights violation as a moral issue—if they did not believe any moral rules were broken, they judged the MNE not to be involved in a violation. When people judged the problem on these terms, they were 26 percentage points more likely to perceive the MNE as being involved in a violation. Another set of justifications was consequentialist and highlighted the effects of the abuse, or lack thereof (3.9%). When respondents evaluated scenarios on consequentialist terms, they were also substantially more likely to judge the MNE as being involved in a violation (26%). A smaller set of responses pointed to specific values, either stating that American companies should (or should not) follow American values abroad (1.2%), or referencing local norms (5.2%).

These results indicate that diffuse informal understandings of human rights and notions of morally appropriate behavior are central to public judgments of MNE conduct. Our findings reveal a gap between American adults’ common views of morally acceptable corporate conduct and the dominant normative positions expressed in soft-law instruments and in the literature. Furthermore, although these results complement theories of attribution of corporate irresponsibility (Lange & Washburn, 2012), they also show that judgments of involvement in human rights violations are a special case of perceptions of unethical corporate behavior.

### Politically Active Respondents

The analyses above reveal the factors that do, and do not, influence public judgments of MNE involvement in human rights abuses. However, many of the individuals in our sample may be unlikely to take action in response to their judgments. It could be that, when we focus on those who are more politically engaged, the effects that we found do not hold. If this is the case, our results may provide a misleading account of when MNEs may face public pressure over their ties to human rights abuses. To investigate this possibility, we analyze a subsample of respondents who are politically active. We create two indices: Political Action Towards Business and Political Action Towards the State (details in Appendix, Sect. Political Action Analysis). We split each of these indices at the median and report results for each group in Fig. 5. For those who score ‘high’ on both indices, our results are substantively similar to the results for the entire sample. Perceptions of MNE involvement in human rights abuses are strongly affected by the relationships between the MNE and the perpetrator, as well as the extent to which the MNE engaged in due diligence, even in groups that are most active in politics. We provide a full account of the minor differences between these groups in the Appendix. Overall, this analysis shows that the substantive patterns of judgment that we find in the general population hold in those who are politically active.

## Conclusion

Scholarly research on business and human rights points to corporations’ efforts to avoid public opprobrium as playing a crucial role in MNEs’ human rights governance (Arnold, 2010; Nolan, 2014). Similarly, the most prominent legal instruments assume a link between broadly held views of corporate responsibility, public reactions to noncompliance with those views, and corporate human rights conduct (Ruggie, 2008a, para. 54). Scholars and practitioners agree that reputational damage can influence profitability, and thus create an incentive for compliance. However, there is stark disagreement as to how likely it is that sufficient reputational damage will occur when MNEs are involved in human rights violations, and there are many doubts about the reliability of public pressure (Clarke & Boersma, 2017; Neureiter & Bhattacharya, 2021; Seidman, 2007). When the public does not judge an MNE to be involved in a violation, we may not be able to rely on reputational pressure alone to punish MNEs when their conduct fails to conform with international human rights standards. Moreover, if MNEs repeatedly escape public backlash when involved in human rights violations, instrumental motivations to prevent harm may erode over time. Therefore, in order to understand when, and to what extent, the threat of reputational damage will underpin human rights governance, it is necessary to determine when the public is likely to conclude that an MNE has been involved in a human rights violation.

This article provides the first systematic empirical account of how the ‘court of public opinion’ judges MNE involvement in human rights violations. Our findings uncover substantial gaps between the judgments of American adults and the prescriptions of soft law and normative arguments. By revealing this gap, this article contributes to our understanding of the limitations of the current international legal framework for business and human rights, which relies heavily on reputational pressure.

We further contribute to the business and human rights literature by offering detailed evidence regarding which factors influence public judgments. We identify specific factors that render an episode unlikely to provoke negative public responses, such as those that involve silent complicity or violations of cultural rights. We also find that the public judges acts committed by suppliers differently from those committed by subsidiaries, in contrast with the results of previous studies (Hartmann & Moeller, 2014). These findings point to contexts in which governance structures that depend on reputation are less likely to induce compliance with international norms. They suggest a need to critically evaluate whether, in light of muted public responses, there will be sufficient pressure on corporations to substantiate a ‘business case’ for compliance in specific circumstances.

By contrast, we also identify factors that render an episode more likely to provoke a negative response, such as those that involve child labor or when an MNE fails to take action based on information gathered during a due diligence process. This last finding suggests that, as MNEs manage human rights risks proactively through due diligence, they also reduce the risk of being pressured by the public. It further suggests that soft law may be reasonably appropriate in imposing due diligence obligations on MNEs, despite the fact that this is also the focus of most of the recent efforts to regulate business human rights conduct at the national level.

This paper makes additional contributions by further linking the study of business and human rights with research that seeks to understand how stakeholders respond to episodes of corporate social irresponsibility (Kölbel et al., 2017; Lin-Hi & Müller, 2013; Wang & Li, 2019). Researchers have called for greater attention to stakeholders’ expectations and understandings of corporate irresponsibility (Nardella et al., 2020). Human rights violations are an important type of irresponsible conduct (Giuliani et al., 2014), and one that has deep legal and normative roots. Theories of corporate social irresponsibility are incomplete if they cannot account for attributions of involvement in human rights violations. Congruent with theoretical work on corporate social irresponsibility, we find that respondents consider the severity of the perceived harm, use moral reasoning, and are attentive to the causal role of the MNE (Lange & Washburn, 2012).

We also report results that are not anticipated in the extant literature on responses to corporate irresponsibility. First, given the centrality of the power to cause and prevent harms, both in the business and human rights literature and in studies of perceptions of corporate social irresponsibility, our expectation was that the public would judge the actions of large and powerful MNEs substantively differently from small ones. According to influential work on perceptions of corporate social irresponsibility, firm size plays an important role in assessments of causality: individuals are more likely to deem a firm causally responsible for an undesirable outcome if the size of the firm and the extent of the harm are congruent (Lange & Washburn, 2012). Our evidence, however, suggests that the public may not be overly concerned with size, or may perceive MNEs of all sizes to be sufficiently powerful: in our study, MNE size has only a minimal influence on judgments of involvement in human rights violations. Hence, our findings support the need for collective efforts to establish causal links when these are not obvious, not just to the organizations involved (Reinecke & Ansari, 2016) but also to the general public.

Second, given the established literature on reputational spillovers (Barnett & King, 2008; Jonsson et al., 2009; Zavyalova et al., 2012), we expected that the industry of the focal MNE would have a substantial effect on public judgments. If this had been the case, it would have indicated that soft-law approaches are most appropriate in high-salience industries. Our evidence is not, however, congruent with this view. We speculate that this may be because the public does not have sufficient knowledge of the human rights records of different industries, and therefore does not generalize from industry to the individual MNEs. Theoretically, this finding suggests scope conditions for theories of reputational spillover—if involvement in human rights violations does not create spillovers across MNEs in the same industry, other categories of unethical behavior may similarly not trigger spillovers. Practically, in light of our results, we should not expect the public to be less demanding towards a solar power company than an oil company, for example.

Third, we uncover factors that have a significant influence on attributions of irresponsibility that have not been widely studied. These include the importance of due diligence in shaping public views, which extends related concepts, such as intentionality, hypocrisy, and rectification (Chen et al., 2020; Clark et al., 2021). Furthermore, our coding of the responses to open-ended questions suggests that the fit between the harm and lay notions of what counts as a human right played a substantial role in people’s judgments. This finding shows that, when it comes to human rights violations, the categorization of harms plays a role beyond factors identified in the literature, such as severity. If activists attempt to frame irresponsible practices as human rights violations, they may fail due to the incongruence with observers’ prior notions of rights. This result has implications both for the study of human rights and for the study of corporate irresponsibility more generally, as schemas for the categorization of harms may condition reactions to corporate misconduct.

Our study has limitations. First, our sample is drawn entirely from a single country. We chose to focus on the U.S. because it is a country where the public is likely to be sufficiently free and powerful to influence the behavior of MNEs. However, the public in other countries may have systematically different views that future research should explore. Future research should analyze not only the views of adults in a range of countries in the global North and South, but also the views of people under 18 years of age. Second, our study only began the process of uncovering the mechanisms underlying public judgments. The coding of the open-ended responses suggests that the use of specific criteria, such as equating involvement with causation, plays a large role in influencing public judgments. Furthermore, the relatively rare reference to legal instruments suggests that, in this context, the law may have less of an expressive power (Sunstein, 1996). Future research can productively analyze the processes by which members of the public make judgments about MNEs and human rights. Third, we do not study how public judgments translate into public pressure on MNEs, either through individual action or through social movement organizations. Research suggests that MNEs value their public standing (Diermeier, forthcoming), that public evaluations of companies can influence consumer behavior (Kam & Deichert, 2020), and that public opinion forms part of political opportunity structures for social movements (Soule, 2009). More work, however, will be needed to completely theorize and empirically examine the process by which the public makes judgments about an MNE’s human rights practices, how those judgments influence pressure on MNEs, and how that pressure ultimately influences corporate practices.

Our study has implications for practice. First, it suggests that human rights organizations and policymakers need to consider the types of episodes that will render public pressure less likely to play an enforcement role. Recognizing these limits can push activists to search for alternative means of enforcement or, when possible, to frame events in ways that take public judgments into account. For instance, arguing that an MNE did not take action to address a potential risk is likely a potent way of shaping public responses, while arguing that an MNE remained silent in the face of an abuse is unlikely to stimulate public pressure. While our findings suggest limits to when the public will judge MNEs to be involved in human rights, our results also indicate that human rights advocates should not be overly preoccupied by perceptions of local norms of acceptable practice: an MNE that violated rights recognized in international legal standards will only have its involvement discounted in a minor way if local norms differ. In addition, our results suggest that human rights advocates can expect the public to put pressure on MNEs of disparate sizes and in a wide range of industries.

Second, our study provides guidance to MNE managers who need to build a ‘business case’ for compliance with human rights norms within their organizations. Our findings suggest that these managers can point to the importance of adhering to internationally recognized norms even when local norms are permissive. Additionally, small MNEs are only given a minor reprieve and are nearly as likely to be judged to be involved in human rights violations as large firms. Finally, when MNEs attempt to prevent potential human rights impacts, they will not only be complying with legal instruments, but they will also be credited by the public. However, managers need to recognize that their firms may face less pressure from the public when the perpetrators are more distant, and when identified risks involve certain types of rights. In such circumstances, instrumental reasons to improve human rights practices may therefore be insufficient, and managers may have to assume obligations with less obvious material benefits.

Our study underscores the importance of taking the workings of the ‘court of public opinion’ seriously, and of understanding when it is more or less likely to contribute to pressure on MNEs to improve their human rights performance. We have built our study using the standards set in soft law, as well as the rich normative literature on business and human rights. We do not set out to resolve ethical debates nor challenge the desirability of policies with our findings. Rather, we identify gaps between policies and ethical arguments, on the one hand, and the general public’s views, on the other. Yet, the public’s view should not be thought of as static, especially as human rights norms evolve over time. Future research should explore how public judgments may shift, so as to better harness public pressure to bring MNE practice closer to normative goals.

## Figures and Tables

**Fig. 6:**
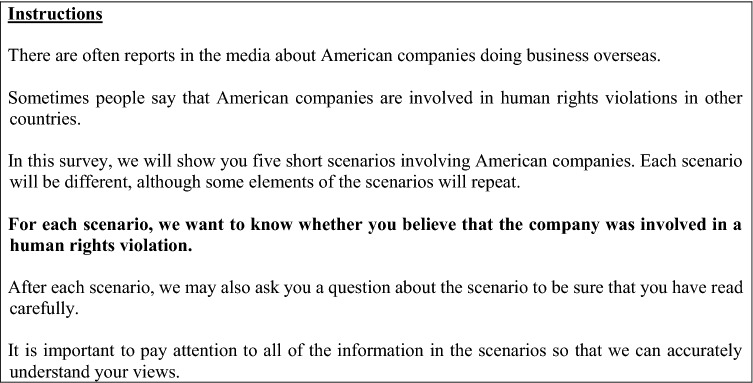
Instructions

## Appendix

This section of the Appendix contains Tables 5, 6, 7, and 8.

**Table 5:** Main results

	*AMCE*
*Relationship (omitted = subsidiary)*	
Supplier	− 0.0753***
	(0.0120)
Beneficial complicity	− 0.102***
	(0.0122)
Silent complicity	− 0.191***
	(0.0129)
*Due diligence (omitted = no due diligence)*	
No action	0.0660***
	(0.0106)
Tried to prevent	− 0.147***
	(0.0116)
*Abuse (omitted = employ child labor)*	
Pay less than living wage	− 0.0521***
	(0.0151)
Contaminate land	− 0.0711***
	(0.0156)
Destroy sacred site	− 0.163***
	(0.0160)
Violently repress protest	− 0.130***
	(0.0154)
Discriminate against minorities	− 0.0545***
	(0.0148)
*Local view (omitted = should not)*	
Can sometimes	− 0.0343***
	(0.00895)
*Size/power (omitted = large)*	
Small	− 0.0338***
	(0.00854)
*Industry (omitted = oil)*	
Clothing	− 0.0182
	(0.0137)
Auto	− 0.00962
	(0.0139)
Solar	− 0.00889
	(0.0139)
Bicycle	− 0.00630
	(0.0138)
Constant	0.829***
	(0.0177)
Observations	12,100
*R*-squared	0.066

Robust standard errors clustered by respondent in parentheses

**p* < 0.05, ***p* < 0.01, ****p* < 0.001

**Table 6:** Comparisons of all industries and abuses

Null hypothesis	*F*	*p*
*Abuse*		
*B*_Living wage_ = *B*_Contaminating_	1.52	0.2184
*B*_Living wage_ = *B*_Sacred Site_	46.96	< 0.001
*B*_Living wage_ = *B*_Protest_	23.66	< 0.001
*B*_Living wage_ = *B*_Discrimination_	0.03	0.87
*B*_Contaminating_ = *B*_Sacred Site_	33.7	< 0.001
*B*_Contaminating_ = *B*_Protests_	13.6	< 0.001
*B*_Contaminating_ = *B*_Discrimination_	1.17	0.28
*B*_Sacred Site_ = *B*_Protests_	3.84	0.05
*B*_Sacred Site_ = *B*_Discrimination_	46	< 0.001
*B*_Protests_ = *B*_Discrimination_	24	< 0.001
*Industry*		
*B*_Clothing_ = *B*_Auto_	0.38	0.54
*B*_Clothing_ = *B*_Solar_	0.45	0.50
*B*_Clothing_ = *B*_Bicycle_	0.73	0.39
*B*_Auto_ = *B*_Solar_	0.00	0.96
*B*_Auto_ = *B*_Bicycle_	0.06	0.81
*B*_Solar_ = *B*_Bicycle_	0.03	0.85

*F*(1, 2419). Not all hypotheses were prespecified (see analysis plan). We report all for completeness

**Table 7:** Political action

	Would never (= 1) (%)	Might (= 2) (%)	Have in the distant past (= 3) (%)	Have in the last year (= 4) (%)
*Action towards business index (mean 8.2, SD 3.2)*				
Signed a petition related to a business	30	47	12	11
Participated in a protest or demonstration about a business’ actions	48	42	6	4
Boycotted certain products for political, ethical, or environmental reasons	32	30	16	22
Deliberately bought certain products for political, ethical, or environmental reasons	34	32	13	21
*Action towards the state index (mean 8.3, SD 3.2)*				
Signed a petition related to a government policy	23	37	18	22
Participated in a protest or demonstration about a government policy	49	36	9	6
Donated money or raised funds for a political activity	44	27	13	16
Contacted or attempted to contact a politician or civil servant to express your views	33	35	14	18

**Table 8:** Codes by abuse

	All vignettes (%)	Child labor (%)	Living wage (%)	Contamination (%)	Sacred site destruction (%)	Protest (%)	Discrimination (%)
Responsive	38.7	34.0	35.4	45.3	36.3	40.0	42.0
Association	7.7	9.5	5.5	5.4	6.7	9.6	10.1
Causal	10.1	6.8	5.5	12.2	12.6	16.3	8.4
Appropriate	3.3	1.4	3.1	2.7	3.0	5.9	4.2
Categorical	19.1	17.7	21.3	14.2	22.2	16.3	23.5
Legal	6.4	13.6	11.0	2.7	4.4	3.0	1.7
Deontological	14.5	20.4	20.1	8.1	15.6	10.4	10.9
Consequential	3.9	4.1	3.1	11.5	0.7	3.0	0.0
American values	1.2	0.7	1.2	0.7	1.5	3.0	0.0
Local norms	5.2	10.9	4.9	6.1	5.2	2.2	0.8
Other	5.5	4.8	4.9	3.4	5.9	6.7	8.4
Number of codable responses	848	147	164	148	135	135	119

Percentage of codable responses by the abuse included in the vignette. Overall, 152 of 1000 responses were not codable

## Robustness Checks

### Attention and Speed

We included four attention checks. All the checks were multiple-choice questions with one correct answer and four filler responses. The first check was presented after the instructions and probed if the respondent recalled the topic of the survey. Respondents who failed this check were reminded of the topic. The remaining three attention checks occurred after the first three vignettes. They asked the respondents to recall one element of the vignette—the size of the focal firm, the industry, or the abuse—presented in random order. In our replication archive, we include analyses of models with all respondents who completed the survey (even those who failed all attention checks, 2795 respondents, *N* = 13,975) and only those who passed all attention checks (1392 respondents, *N* = 6960). As expected, the effect sizes are larger for attentive respondents, but the overall pattern of responses does not change. In the replication archive, we also include analyses of the respondents removing those who were ‘speeders,’ defined as the 5th or 10th percentiles in survey speed. Again, we see the expected larger effect sizes for attentive respondents, but the same overall pattern of results.

### Order of Presentation

We did not randomize the order of all elements of the vignettes in order to maintain the narrative flow. We did, however, randomize the order of the last part of the vignette—with half the respondents seeing the due diligence information before local views, and half seeing the vignettes in the opposite order. In the replication archive, we include analyses of order effects. We find that the results are largely unchanged by order of presentation.

### Rounds

Our method for estimating the AMCEs assumed that there are no carryover effects, meaning that the results are stable across the rounds (i.e., the effects of treatments in the first round are the same as those in the later rounds). In our replication archive, we report the results of our main analyses by round to investigate carryover effects. We include the most conservative approach to carryover effects, which involves only using data from the first round.

While these estimates are less precise, we find the same support for all analyses, with one exception (the difference in the effect of ‘supplier’ or ‘subsidiary’ is not statistically significant).

### Weighting

Although our sample closely resembles the U.S. population, it is underrepresented in people with less than a high school education and people who identify as Hispanic. Such unrepresentativeness will only bias our results if these attributes interact with our treatments. As an additional robustness check, we estimate our main models weighting our sample to approximate the U.S. population more closely; we find that the results are unchanged. We include these results in the replication archive.

## Political Action Analysis

To measure each respondent’s past political action, we crafted a set of questions drawing on a similar block in the General Social Survey (GSS). To create the indices of political action, we took the sum of responses to each block of four questions (Action Towards Business, Cronbach's *α* = 0.81; Action Towards the State, Cronbach’s *α* = 0.79). For those who report high (above the median) levels of political action towards firms, in the last year: 37% report boycotting, 36% report purchasing a product because of the company’s labor or environmental record, 18% report signing a petition, and 7% report participating in a protest. For those who report high levels of political action towards the state, in the last year: 37% report signing a petition, 32% contacting an official, 28% raising funds or donating money, and 11% participating in a protest.

Figure 5 in the main text reports the results by group. There is a difference in the overall fit of the models comparing high/low on political action towards business (omnibus *F* (1,2419) = 2.86, *p* < 0.001) and high/low on political action towards the state (omnibus *F*(1,2419) = 2.52, *p* < 0.001).

Examining specific treatments helps us to understand these differences. The effect of silent complicity is more negative on those who have taken more political action towards firms (ACIE =  − 0.057, *p* = 0.029, 95% CI [− 0.11, − 0.006]) and the state (ACIE =  − 0.072, *p* = 0.005, 95% CI [− 0.12, − 0.022]). Those with histories of greater political action towards business are even more strongly affected by firms taking action to prevent an abuse (ACIE =  − 0.065, *p* = 0.006, 95% CI [− 0.11, − 0.019]), but we do not find the same difference in effect for those who are more politically active towards the state (ACIE =  − 0.021, *p* = 0.37, 95% CI [− 0.067, 0.025]). While these findings reveal differences, they do not suggest that the judgments of politically active people are more aligned with soft law and normative arguments.

In terms of abuses, we observe less of a difference between child labor and violent repression of protests for both those who are more politically active towards business (ACIE = 0.087, *p* = 0.005, 95% CI [0.026, 0.015]) and the state (ACIE = 0.074, *p* = 0.017, 95% CI [0.013, 0.13]). This result is expected, considering that the politically active subsamples are more likely to have taken part in protests themselves. Finally, we observe some differences in the role of industry across the two groups: those who score higher in political action are more likely to perceive that an oil company is involved in a human rights abuse than clothing or automobile manufacturers. Yet, the results of neither group fit the overall pattern that would be expected if people used the human rights record of an industry to make inferences about MNE involvement.

## Open-Ended Responses

### Coding Guide

To develop our codes, each co-author read a small sample of responses and inductively coded them based on themes that emerged. We iterated this process, identifying themes and relating them to the literature. Over time, two broad areas of interest emerged: evaluations of the relationship and evaluations of the abuses. We then created a set of definitions for the codes (explained below).

We then drew a new random sample of 500 respondents and the authors hand coded the responses to the two open-ended questions, providing data for a total of 1000 responses. Where there was uncertainty in a code, all three co-authors reviewed the code and came to a consensus. We allowed for multiple codes per response. Our definitions for each code and decision rules are reported below.

### Evaluation of Relationship

- **Causal role:** These responses point to a perceived causal role, or lack of causal role, in the abuse. The assessment can go in either direction, but the key is that the respondent’s criterion is causality. We include causal role when the response references “control,” which implies causal action by the principal. We take pronouns like “they” as referring to the MNE.
- **Responsiveness:** How the MNE responds to potential or actual human rights abuses. This includes a lack of responsiveness, in which the MNE is judged to be indifferent to the harm to others. Included here are statements that the company had the capacity to act, but did not. It also includes actions that MNEs take to prevent an abuse. These responses differ from the ones above in that they do not focus on causation of the abuse, but how the MNE acts within the context of its relationship.
- **Association:** These statements define involvement simply through association. This includes doing business in a country that has a poor human rights record. This also includes benefitting from the actions of others.
- **Appropriate role:** These statements point to the appropriate role of business. This includes statements that it is the government/state, not business, that is responsible for human rights protection.

### Evaluation of Abuses

- **Categorical Fit:** Responses stating that something is, or is not, a human rights violation, without elaboration of the reasoning.
- **Legal:** Responses about what counts as a human rights violation explicitly or implicitly referencing legal structures. This includes statements that action is “illegal” or that cite what respondents believe are laws (e.g., businesses have to pay the minimum wage). These references can be both positive (it is illegal, therefore a human rights violation) or negative (nothing illegal about the action, therefore not a human rights violation).
- **Deontological**: These responses evaluate the abuse in terms of universal norms or values. They suggest the use of moral criteria for decision making.
- **Consequential:** These statements talk about the consequences of the abuse.
- **“American” values**: Responses explicitly note the values of the home country—in this case, the United States. They explain their judgments by virtue of shared American values.
- **Local norms:** Responses that reference what they believe are norms of appropriate behavior in the country where the abuse took place.

### Additional Codes

- **Other:** An interpretable response that does not fit one of the above codes.
- **Not Codable**: A response that cannot be interpreted to be coded.

## Instructions

See Appendix Fig. 6.

## Data availability

Upon publication, all data and code will be posted in the OSF public data repository. The full transcript of the survey is included in the pre-registration, which has been posted on OSF: https://osf.io/56dnp/

## Footnotes

1 An important part of the literature on business and human rights argues that MNEs possess human rights obligations that go beyond a narrow interpretation of the most prominent soft-law instruments (Santoro, 2009; Wettstein, 2009; Wood, 2012). Throughout this paper, we will refer to normative arguments in the business and human rights literature to discuss the nature and scope of MNE responsibility, in addition to the provisions contained in soft-law instruments; these arguments mostly support, but sometimes go beyond, the legal framework. This approach allows us to shed light on a broader range of questions, and to respond to the evolving nature of the legal framework.

2 There is a small literature on the general public’s view of human rights (Hertel et al., 2009) and human rights organizations (Ron et al., 2017), yet it does not focus on perceptions of business.

3 The registration is posted at OSF: https://osf.io/56dnp/ All data and the statistical code to replicate the analyses will be made publicly available in the same OSF location upon publication.

4 The survey was approved by the submitting authors’ departmental Research Ethics Committee.

5 While we expected the sample from Dynata’s pool to be representative, we also used quotas to ensure that our final sample closely matched the U.S. population in terms of age, gender, region, race/ethnicity, and education.

6 Research demonstrates that including five tasks should not bias results (Bansak et al., 2018). In all analyses, we cluster standard errors by respondent.

7 Our design was fully randomized (eliminating the need for exclusions).

8 In order to maintain the logical flow of the vignettes, we did not randomize the order of all elements; respondents were always told the MNE’s size and industry before being confronted with the abuse. However, the order of the final two elements of the vignettes (local view and due diligence) did not create a logical constraint. Therefore, we randomized the presentation of each of these elements by respondent.

9 The number for oil includes the oil, gas, and coal industries. The database does not disaggregate these industries.

10 Each round of the survey used a different letter for the company, running A through E.

11 The survey included four attention checks to increase engagement and screen out non-engaged respondents (described in Appendix, Sect. Attention and Speed). We excluded respondents who did not pass at least two attention-check questions.
